# Th2 mRNA gene expression analysis separates Prurigo nodularis into two immune signature groups

**DOI:** 10.1111/jdv.20812

**Published:** 2025-07-02

**Authors:** Sonja Ständer, Emma Guttman‐Yassky, Gil Yosipovitch, Henning Wiegmann, Dieter Metze, Madeline Kim, Ester Del Duca, Kent Bondensgaard, John F. Paolini, The LOTUS‐PN Investigators Group, David Pariser, Gil Yosipovitch, Adam Friedman, Timothy Berger, Brian Kim, Jonathan Silverberg, Maria Alora‐Palli, Swarna Ekanayake‐Bohlig, Sonja Ständer, Jacek Szepietowski, Elke Weisshaar

**Affiliations:** ^1^ Pruritus Medicine Section, Department of Dermatology, and Center for Chronic Pruritus (KCP) University Hospital of Münster Münster Germany; ^2^ Department of Dermatology Icahn School of Medicine at Mount Sinai New York City New York USA; ^3^ Dr. Phillip Frost Department of Dermatology & Cutaneous Surgery, Miller School of Medicine University of Miami Miami Florida USA; ^4^ Kiniksa Pharmaceuticals Corp Lexington Massachusetts USA

**Keywords:** chronic prurigo, neuroimmune itch pathways, OSMR pathway, prurigo nodularis, pruritus

## Abstract

**Background and Objectives:**

Prurigo nodularis (PN) is a severe, intensely pruritic subtype of chronic prurigo for which the molecular basis and interplay between pathophysiologic mechanisms and clinical manifestations are poorly understood.

**Methods:**

LOTUS‐PN was a longitudinal observational study that included 54 participants from 11 centers and was designed to improve disease understanding. Protein expression, histology and RNA analyses were performed.

**Results:**

Histologically, all patients had typical PN characteristics. Immunohistochemically, and on the mRNA level, expression in lesional versus non‐lesional samples for IL‐31 (*p* < 0.01) was significantly higher. In addition, immunohistochemistry showed higher expression of IL‐31RA (*p* < 0.05), OSM (*p* < 0.05) and OSMRß (*p* < 0.01). PN lesional skin showed higher expression of Th2 and Th17/Th22 pathway markers. A correlation analysis of gene expression highlighted two groups of intra‐marker correlations dividing the Th2 markers, the first containing barrier and regulatory genes and the other containing markers of general inflammation that correlated negatively and positively with Th17/Th22 genes, respectively. Correlations between gene expression and NRS scores were weak and involved genes associated with general inflammation ( *MMP12* ) and Th2 ( *IL10* , *CCL18* , *IL4R* ).

**Conclusions:**

Our study confirms the role of Th2 signature in PN and the dominant role of IL31. Correlation analysis of gene expression showed differing relationships between subsets of the Th2 axis with Th1 and Th17/Th22 markers, highlighting the potential of different components of the Th2 pathway to interact with and modulate other immune axes.

## Graphical abstract

Protein expression, histology and RNA analyses were performed from patients with prurigo nodularis (PN). All patients had typical PN characteristics. Lymphocytes/monocytes demonstrated more frequent positive staining signals in lesional versus non‐lesional samples for IL‐31, IL‐31RA, OSM and OSMRb. IL‐31, Th2 and Th17/Th22 pathway mRNA levels were higher in lesional biopsies.

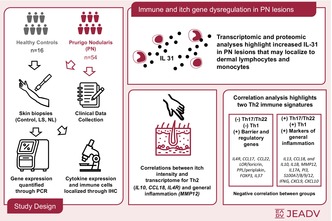

**Why was the study undertaken?**

- PN molecular mechanisms remain poorly characterized across diverse patient cohorts.

**What does this study add?**

- This analysis of PN transcriptomic and immunohistochemical profiles provides key insights into disease pathogenesis. Biomarker data confirm the relevance of IL‐31, OSM and their receptors, reinforcing the role of Th2 cytokines in PN.

**What are the implications for disease understanding and clinical care?**

- Targeting IL‐31 and OSM may offer therapeutic benefits by addressing pruritus and inflammation in PN.

## INTRODUCTION

Prurigo nodularis (PN), a subtype of chronic prurigo,^1^ is characterized by multiple intensely pruritic nodules.^2^, ^3^, ^4^ The severe pruritus contributes to poor sleep and reduced quality of life.^1^

Limited information is currently available regarding the molecular basis of PN and the connection between pathophysiology and pruritus intensity. A thorough understanding of molecular mechanisms that drive PN pathogenesis is crucial for the development of effective therapies. Recent data implicate Th2‐associated cytokines such as interleukin‐4 (IL‐4), IL‐13, IL‐31 and oncostatin M (OSM) as important mediators of pruritus and responsible for PN typical epidermal hyperplasia, inflammation and fibrosis.^2^, ^5^, ^6^, ^7^, ^8^, ^9^ While PN has been associated with underlying comorbidities (e.g. atopic predisposition HIV, end‐stage renal disease),^10^, ^11^, ^12^, ^13^ such relationships are not ubiquitous in PN, and the role of concomitant disease remains unclear.^14^

LOTUS‐PN (Longitudinal Trial to Understand Symptomatology in Prurigo Nodularis [chronic prurigo]) is a longitudinal observational study of PN. LOTUS‐PN addresses a comprehensive scope of clinical and pathophysiologic factors related to PN with the goal of enhancing understanding of histopathological and relevant biomarkers associated with PN. This paper describes an analysis of baseline findings from the LOTUS‐PN study population that pertain to correlations of mechanistic biomarkers with clinical measures of disease severity.

## MATERIALS AND METHODS

### Study design

LOTUS‐PN, initiated in September 2017, was a prospective, longitudinal study that examined PN patients in routine clinical practice. The study was conducted at 11 sites across the United States, Germany and Poland. Principal investigators are listed in Acknowledgements. No medications were administered as part of the study protocol. Patients were managed with standard of care treatment and followed up per routine medical care.

Eligible patients were ≥18 years of age with a new/previously established PN diagnosis as confirmed by a physician investigator experienced in PN. Sample size was estimated based on previous studies and patient availability.^15^ Exclusion criteria included recent/planned participation in a clinical trial, prior treatment with an immunosuppressive or biologic agent within five half‐lives or 3 months prior to enrollment and unwillingness to participate in skin biopsies.

Baseline assessment included documentation of patient demographics, medical history, vital signs, height/weight/body mass index, PN disease severity based on the Prurigo Activity and Severity Score (PAS), medical photography of the lesion(s), blood samples for biomarkers/genomic testing and biopsies of PN lesions. Blood samples were shipped to a centralized laboratory for analysis. Lesion types and distribution were assessed with anonymized whole‐body photographs (Canfield Scientific). During the baseline assessment, patients were provided with e‐diaries to document their responses to assessments.

Patients recorded daily average and peak itch intensity within the prior 24 h using a numeric rating scale (NRS) ranging from 0 (no itch) to 10 (worst imaginable itch). Patients also completed the Itch Controlled Days questionnaire (ICD Version 12) describing the nature of itching and sleep disturbances during the previous 24 h.

Pruritus was also assessed using the 5‐D itch scale, which evaluates five domains (duration, degree, direction, disability and distribution) over a recall period of 2 weeks, with possible total scores ranging from 5 (no pruritus) to 25 (most severe pruritus).^16^

The study was conducted in accordance with the Declaration of Helsinki and the International Conference on Harmonization Good Clinical Practice guidelines. All participants provided written informed consent prior to study participation and were informed that study participation would not change or affect their usual care. A separate, optional consent form was provided for genomic testing. The study protocol and amendments were approved by Sterling Institutional Review Board (central IRB) and local IRBs and Ethics Committees where applicable.

### Skin biopsies

Following photography, up to three 4‐mm punch biopsies were obtained. One sample was acquired from a non‐lesional location, and up to two samples were acquired from lesional locations. The best representative lesion was selected for biopsy. Samples were split in half for a subset of samples, and sites were given the option to split biopsies using the procedure described by Pariser.^17^ One half was placed in paraformaldehyde and used for histologic examination and immunohistochemistry analysis. The other half was placed in RNAlater® Stabilization Solution and used for RNA extraction and biomarker analysis. If examples of insufficient RNA yield and poor quality were identified, the splitting process was removed. The subset of samples that had not been split was placed in RNAlater® Stabilization Solution for RNA extraction.

### Histology

Tissue sections were stained with H&E according to standard protocol. The paraformaldehyde‐fixed biopsies were treated with ascending sucrose concentrations, then stored at −20°C without liquid. Frozen samples were then dehydrated and paraffinized. From the paraffin blocks, 5‐μm sections were prepared with a microtome and transferred to a slide. H&E staining was performed with a Tissue‐Tek® Prisma® Automated Slide Stainer (Sakura Finetek USA, Torrance, CA). In total, 21 pathologic epidermal and dermal characteristics were evaluated (Figure S1). Each characteristic was determined as being either absent or present.

### Immunohistochemistry

Immunohistochemical staining was used to examine the expression of IL‐31, IL‐31RA, OSM and OSMRβ in the epidermis, smooth muscle, endothelia, lymphocytes/monocytes, dermal nerve fibres and fibroblasts. Each expression was rated negative, positive or strong positive.

Cryosections were previously permeabilized by treatment with proteinase K for 10 min at room temperature (RT). Sections were then rinsed (Labline wash buffer DCS WL 583 C2500, 1:20 with distilled water; DCS, Hamburg, Germany) and transferred to a staining chamber. For each section, 100 μL of primary antibody was added at specific dilution and incubated for 45 min at RT. After incubation, sections were rinsed 2 × 3 min and incubated with 1 drop each of DCS Polylink horseradish peroxidase (HRP) Kit PD000RP for 15 min at RT. After rinsing again 2 × 3 min, 1 drop of streptavidin HRP (DSC Polylink HRP Kit PD000RP) was added to each section and incubated for 15 min at RT. After incubation, the sections were rinsed again and incubated for 15 min at RT in 3‐amino‐9‐ethylcarbazole (AEC, AC 1310100 AEC 2 component kit DCS). Sections were counterstained with hemalaun. The following antibodies and dilutions were used: α‐IL31 (PA5‐20220, Thermo Fisher) 1:800; α‐IL31RA (ab113498, Abcam) 1:200; α‐OSM (PA5‐76861, Thermo Fisher) 1:200; α‐OSMR (10982‐1‐AP, Proteintech) 1:40.

### Gene expression

Tissue from lesional and non‐lesional biopsies of patients with PN, and control samples from healthy volunteers, were used to define a transcriptional profile for PN skin. RNA was extracted from FFPE blocks, and Taqman Low Density Array (TLDA) quantitative reverse‐transcriptase–polymerase chain reaction RT‐PCR (qRT‐PCR) was performed to determine gene expression profiles for 46 genes indicative of inflammatory responses and barrier function (Table S1). These 46 genes were selected for their pathogenic role in atopic dermatitis (AD), a better characterized inflammatory skin disease with similar Th2 dominance.^18^, ^19^ Reverse transcription to cDNA was carried out using High‐Capacity complementary DNA reverse transcription (Thermo Fisher, Waltham, MA). TLDA cards (Thermo Fisher Scientific, Waltham, MA) were used for qRT‐PCR. Eukaryotic 18S rRNA was used as an endogenous control. In each participant, expression levels from RT‐PCR were normalized to the housekeeping gene ribosomal protein lateral stalk subunit P0 (RPLP0) by negatively transforming the Ct values to –dCt, obtaining an equivalent to log2 scale expression values (reporting unit: log2 [Expression/RPLP0]). Samples were considered positive for target gene expression if the cycle threshold value was <35, and for each gene, missing values were imputed with 20% of the minimal unlogged expression over the detection limit across all samples.

### Statistical analysis

There was no formally stated or statistically powered a priori hypothesis for this study, and findings were generally descriptive in nature. Statistical analysis of histologic and immunohistochemical assessments was performed using the McNemar test for paired categorical samples. Normalized gene expression profiles were modelled by linear regression analysis. Correlations of clinical NRS scores of itch intensity and mRNA levels in lesional and non‐lesional skin were evaluated with Pearson correlation coefficients, with visualization within heatmaps performed with k‐means clustering. *p*‐values are nominal and not adjusted for multiplicity. Unsupervised hierarchical clustering of average gene expression for heatmap visualization was performed with the McQuitty agglomeration algorithm. All statistical analyses were performed using R programming language.

## RESULTS

### Patients

The study enrolled 54 adult patients with PN (United States [*n* = 34, 63.0%], Germany [*n* = 15, 27.8%] and Poland [*n* = 5, 9.3%]). The mean (SD) age was 53.8 (±12.9) years, and most (57.4%) were female and white (64.8%) (Table 1). Lesional and non‐lesional skin biopsies were obtained at enrollment. Histology and RNA analyses were performed on 53 samples due to a lost sample at one of the study sites. Control biopsies from 16 healthy individuals, obtained from the tissue bank of the Guttman laboratory, were included as a benchmark.

**TABLE 1:** Patient demographics and disease characteristics.

	*N* = 54
Age, years	
Mean (SD)	53.8 (12.92)
Min, max	19, 74
Sex, *n* (%)	
Male	23 (42.6)
Female	31 (57.4)
Reported ancestry and ethnicity, *n* (%)	
American Indian or Alaska Native	1 (1.9)
Asian	1 (1.9)
Black or African American	15 (27.8)
Hispanic or Latino	2 (3.7)
Not Hispanic or Latino	51 (94.4)
White	35 (64.8)
Other	2 (3.7)
Unknown	1 (1.9)
Country of residence, *n* (%)	
United States	34 (63.0)
Germany	15 (27.8)
Poland	5 (9.3)
Estimated number of lesions, *n* (%)	
1–19 lesions	14 (26)
20–100 lesions	30 (56)
>100 lesions	10 (19)
Time since PN signs/symptoms started, years, mean (SD)	10.4 (11.35)
Time since PN diagnosis, years, mean (SD)	5.1 (10.08)
Age at diagnosis, years, mean (SD)	48.8 (14.87)

Abbreviations: PN, prurigo nodularis; SD, standard deviation.

### Disease characteristics and pruritus severity

Disease severity as assessed at baseline using the Prurigo Activity and Severity Score (PAS)^20^ revealed that most patients (56%) had 20–100 pruriginous lesions (Table 1). Patients reported itching on numerous parts of their body during the preceding two‐week period, with a mean (SD) (*n* = 47) 5‐D Itch Total Score of 15.8 (±3.3). Patients described the impact of itching on their sleep over the preceding 2 weeks as ‘never affects sleep’ (*n* = 6, 13%), ‘occasionally delays falling asleep’ (*n* = 10, 21%), ‘delays falling asleep and occasionally wakes me up at night’ (*n* = 14, 30%) and ‘delays falling asleep and frequently wakes me up at night’ (*n* = 11, 23%).

Baseline pruritus was rated by patients as mild (29.8%), moderate (46.4%), severe (15.7%) or very severe (5.7%). On the NRS, the mean (SD) weekly score for the pruritus intensity was 5.0 (±1.1), and the mean (SD) weekly score for the worst pruritus intensity was 6.1 (±1.2). Sleep disturbances in the prior 24 h due to pruritus were indicated on e‐diary entries by 45.6% of patients during the first week of the study.

Over 50% of patients described an impact of their itching on their sleep over the preceding 2 weeks on the 5‐D Itch questionnaire.

The most prevalent comorbid conditions among patients participating in the study included hypertension (38.9%), high cholesterol (18.5%), hypothyroidism (18.5%), AD (16.7%), asthma (14.8%), type 2 diabetes (11.1%) and allergic rhinitis (11.1%). The medical conditions which were common and frequently identified by the physician investigators as underlying conditions for their patient's PN were hypothyroidism (19% of patients), AD (17% of patients) and depression (20% of patients). Conditions that were not common but frequently identified as the underlying condition of PN were HIV/AIDS (6% of patients), hepatitis C (4% of patients), chronic renal failure (4% of patients) and bipolar disorder (4% of patients). Investigators did not identify or report an underlying medical condition for PN for 35 of the 54 participating patients.

### Histology

**FIGURE 1:**
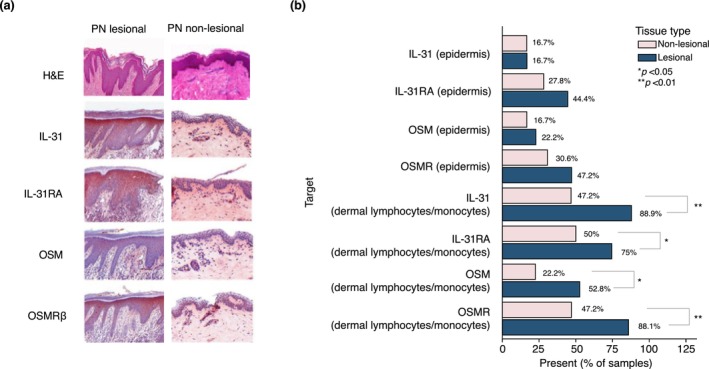
Expression of IL‐31, IL‐31RA, OSM and OSMRß protein in epidermis, endothelia, smooth muscle, lymphocytes/monocytes, dermal nerve fibres and fibroblasts. (a) H&E and immunohistologic assessment of IL‐31, IL‐31RA, OSM and OSMRβ expression in various skin targets. Original Magnification ×100. (b) Immunohistochemical evaluation of IL‐31, IL‐31RA, OSM and OSMRβ in epidermis and lymphocytes/monocytes of lesional (teal bars) and non‐lesional (purple bars) skin of patients with PN. Original Magnification ×100. *p < 0.05. **p < 0.01. H&E, haematoxylin–eosin; IL‐31, interleukin‐31; IL‐31RA, interleukin‐31 receptor subunit alpha precursor; NL, non‐lesional; OSM, oncostatin M; PN, prurigo nodularis.

Histologic data were evaluated from 53 patients with PN. Haematoxylin–eosin (H&E) staining revealed significant differences in lesional skin (LS) versus non‐lesional skin (NLS), including characteristics typical of PN, such as epidermal hyperplasia, fibrosis and inflammation (Figure 1). LS in comparison to NLS showed significantly more frequent epidermal hyperplasia (*p* < 0.05), dermal vertically arranged collagen fibres (60% vs. 0%), increased numbers of collagen fibres (86% vs. 0%), fibroblasts (82% vs. 0%), capillaries (74% vs. 0%) (all *p* < 0.0001) and more pronounced perivascular infiltrate (94% vs. 26%), interstitial inflammatory infiltrate (30% vs. 0%), as well as superficial (94% vs. 26%) and deep (34% vs. 0%) infiltrate (all comparisons, *p* < 0.001) (Figure S1). In the composition of the inflammatory infiltrate, neutrophils (26% vs. 2%; *p* < 0.01) and eosinophils (34% vs. 12%; *p* < 0.05) were significantly more abundant in LS versus NLS in PN. Differences in the presence/absence of infiltrating macrophages, plasma cells and mast cells were not significant (data not quantitatively analysed; not shown).

Expression of IL‐31, IL‐31RA, OSM and OSMRß protein was evaluated in epidermis, endothelia, smooth muscle, lymphocytes/monocytes, dermal nerve fibres and fibroblasts using immunohistochemical staining; most of these cutaneous structures and cells showed no differences in expression between LS and NLS. However, interstitial lymphocytes and monocytes of the dermis demonstrated significantly more frequent positive signals in LS versus NLS for IL‐31 (88.9% vs. 47.2%; *p <* 0.01), IL‐31RA (75% vs. 50%; *p* < 0.05), OSM (52.8% vs. 22.2%; *p* < 0.05) and OSMRß (86.1% vs. 47.2%; *p* < 0.01) (Figure 1a,b).

### Gene expression

**FIGURE 2:**
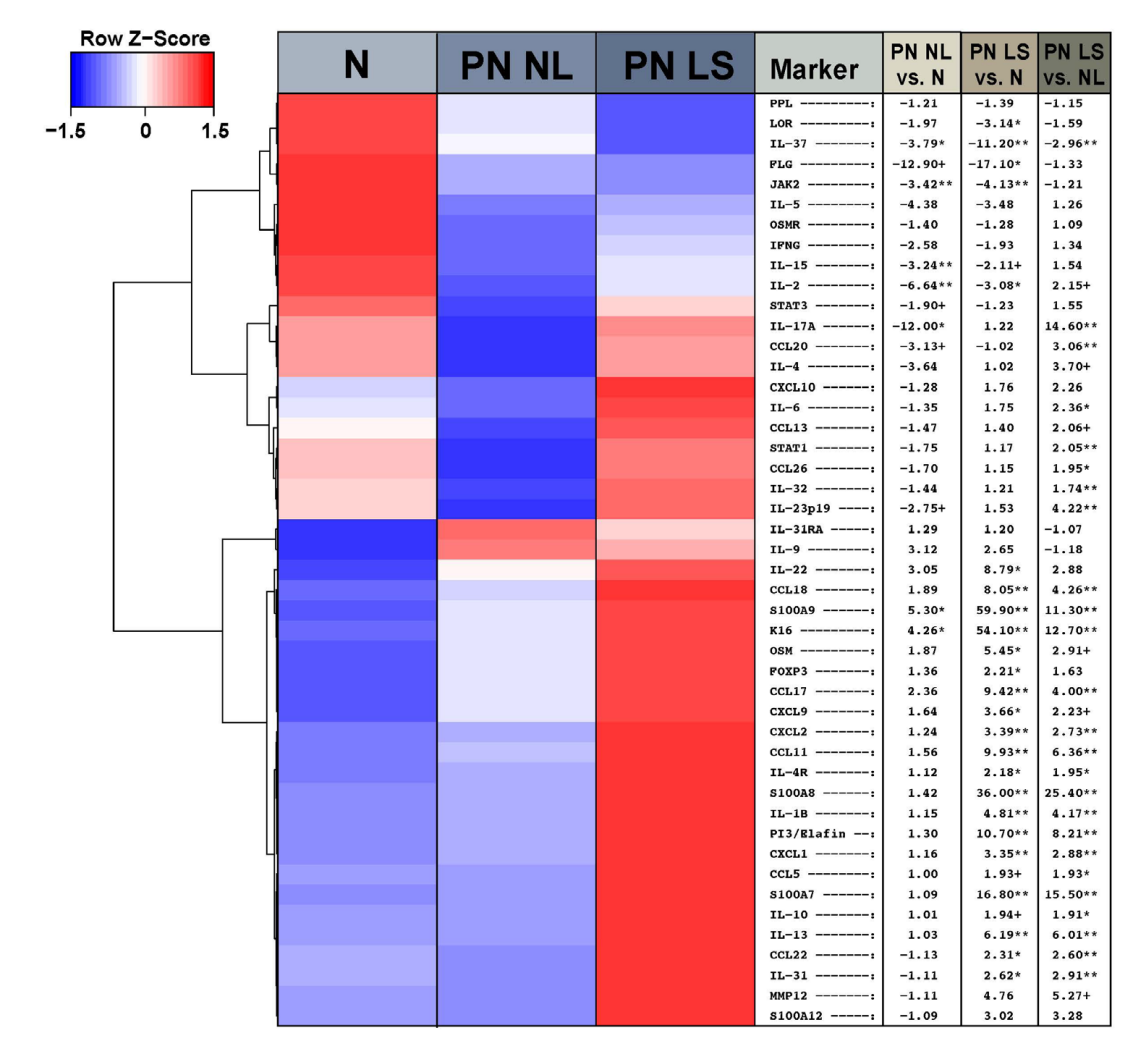
Heatmap of mean immune‐ and barrier‐gene expression measured by RT‐PCR in healthy controls, non‐lesional PN and lesional PN. The adjacent table shows expression fold‐changes, from left to right, in non‐lesional PN versus healthy controls, lesional PN versus heathy controls and lesional versus non‐lesional PN. LS, lesional; NL, non‐lesional; PN, prurigo nodularis. +*p* < 0.1; *p < 0.05; **p < 0.01.

Gene expression analysis identified a distinct profile in PN‐LS biopsies compared to NLS and healthy control samples; NLS and healthy controls were largely similar (Figure 2). Th2 and Th17/Th22 pathway mRNA levels were found to be significantly higher in LS. Th2‐associated *IL4R*, *IL13*, *CCL13*, *CCL17* and *CCL22* were significantly upregulated in LS compared to NLS and healthy controls (*p* < 0.05), as was *IL31*, in line with immunohistochemical results in lymphocytes/monocytes. Within the Th17 and Th22 axes, *IL22* and *PI3*/elafin were significantly increased in LS. Significant upregulation of genes associated with IL‐22‐driven epidermal hyperplasia (*S100A7*, *S100A8*, *S100A9*) were also observed in LS versus healthy controls and NL for hyperkeratotic skin conditions.^21^ Increases in Th1‐related gene products were comparatively modest. Additionally, significant decreases in *FLG*/filaggrin and *LOR*/loricrin were observed in LS while modulation of *PPL*/periplakin was less pronounced.

### Correlation analysis of gene expression and pruritus intensity

**FIGURE 3:**
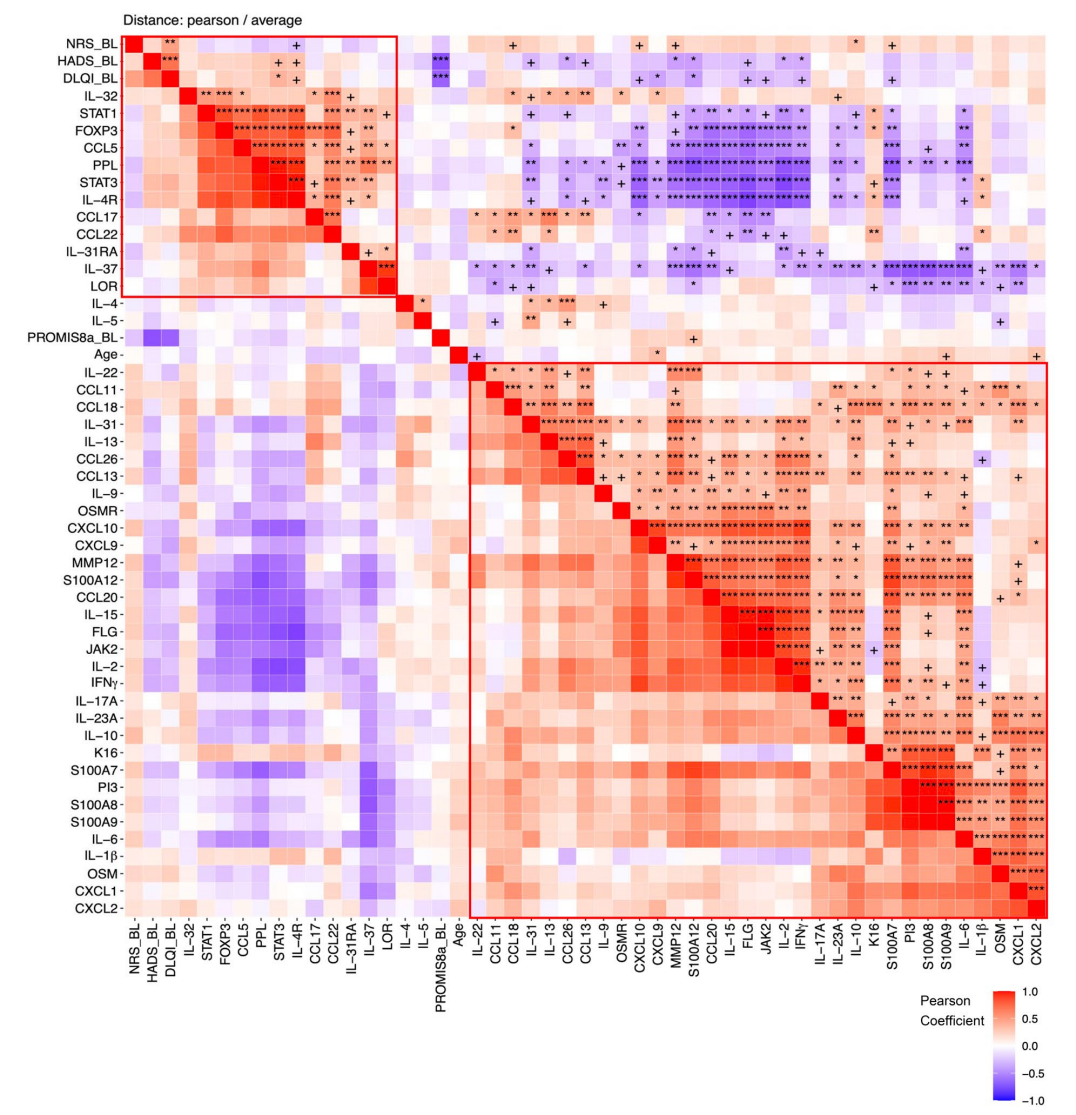
Pearson correlation analysis of clinical NRS scores of itch intensity at baseline and mRNA levels of lesional skin biopsies. Positive correlations (significant or approaching significance) were observed between NRS scores and Th17, Th1, pruritus and general inflammation pathways. Two major correlation clusters are outlined in red. Th17 (*S100A*s), Th1 (*CXCL9*, *CXCL10*), Th2 (*IL‐13*, *CCL11*), Itch (*IL31*) and General inflammation (*MMP12*) markers were significantly associated and clustered. +*p* < 0.1. *p < 0.05. **p < 0.01. ***p < 0.001. CCL, chemokine ligand; CXCL, chemokine (C‐X‐C motif) ligand; IL, interleukin; MMP12, matrix metalloproteinase‐12; NRS, numeric rating scale; Th, T helper.

Pearson correlation analysis of gene expression and clinical NRS scores for pruritus intensity showed strong, positive correlations between Th17/Th22, Th1 and Th2 markers in both LS (Figure 3) and NLS (Figure S2). Across tissue types, Th2 markers were divided between two distinct immune signature groups formed by these intra‐marker correlations.

The first group contained *IL4R*, *CCL17* and *CCL22*, as well as barrier genes *LOR*/loricrin and *PPL*/periplakin and regulatory genes *FOXP3* and *IL37*. Many genes within this first group correlated negatively with markers within the second group, which included Th2‐associated *IL13*, *CCL18* and *IL10*, markers of general inflammation (*IL1B* and *MMP12*), Th17/22 markers (*IL17A*, *PI3*, *S100A7/8/9/12*) and Th1‐associated markers (e.g., *IFNG*, *CXCL9* and *CXCL10*). Markers within a given cluster generally associated strongly and positively with each other and negatively with members of the other cluster. Notably, Th2 markers within the first cluster containing barrier and regulatory markers were associated negatively with Th2, Th17/22, Th1 markers within the second group.

Correlations between gene expression and NRS scores were overall weak, though some associations were observed with genes associated with itch (*IL31*), general inflammation (*MMP12*), Th2 (*IL10*, *CCL18*, *IL4R*), Th1 and neutrophils (*CXCL10*) and epidermal hyperplasia (*S100A7*) (Table 2).

**TABLE 2:** Analyses of correlations between mRNA gene expression and NRS scores at baseline.

	Weekly worst NRS correlation coefficient, Pearson *R*	Weekly worst NRS, *p*‐value
IL‐10	0.33	0.0359
CCL18	0.30	0.0599
CXCL10	0.29	0.0685
MMP12	0.29	0.0700
IL‐4R	−0.27	0.0888
IL‐31	0.27	0.0915
S100A7	0.26	0.0999

Abbreviations: CCL, chemokine ligand; CXCL, chemokine (C‐X‐C motif) ligand; IL, interleukin; MMP12, matrix metalloproteinase‐12; NRS, numerical rating scale.

## DISCUSSION

LOTUS‐PN was conducted to investigate the disease burden and pathophysiology of PN. The principal objectives were to collect patient demographics and medical history, patient‐reported outcomes, clinical (physician‐reported) outcomes and mechanistic biomarkers in patients with PN. The LOTUS‐PN cohort reported substantial burden related to itching at baseline, including half of the patients reporting sleep disturbances due to itching. Two‐thirds of the cohort rated the severity of itching as moderate to very severe. These data reflect a high unmet therapeutic need, as inclusion into the study was agnostic of antipruritic therapy. The most prevalent comorbid conditions among patients with PN were hypertension, high cholesterol, hypothyroidism, AD, asthma, Type 2 diabetes and allergic rhinitis. Of note, AD was not a prominent feature of the patients, with only 16.7% having AD.

The skin of all patients displayed typical histopathological PN characteristics, including epidermal hyperplasia, fibrosis and inflammatory infiltrate in the dermis in line with a previous analysis which demonstrated the dominance of lymphocytes and macrophages/monocytes in the infiltrate.^22^ Our findings indicate that lesional skin expresses significantly higher markers of the IL‐31 pathway (i.e. IL‐31, IL‐31RA, OSM and OSMRß) versus NLS, on the transcriptome and protein level in line with previous reports.^23^, ^24^ In a US cohort, increased dermal expression of IL‐31 and IL‐31RA in PN lesions was closely correlated with itch intensity in PN.^6^ In our cohort of patients, while itch NRS was not significantly correlated with IL31 expression, the association did trend positive. This may suggest a more complex role of IL‐31 in perpetuating itch. Future studies may clarify the influence of factors such as disease duration on this relationship. In addition, further studies are needed to clarify the role of ethnicity. However, the clinical relevance of the IL‐31 signalling pathway has been substantiated in studies with IL‐31 blocking‐targeting drugs, which significantly improved pruritus in patients with PN independent of ethnicity, age, gender or comorbidity.^2^, ^25^ Further, our study found significantly increased positive signals for OSM and OSMRß staining in lymphocytes and monocytes from LS samples of patients with PN. This is an important finding as OSM, like IL‐4, has the function to sensitize neurons and enhance the response to pruritogens. OSM has been found in a previous PN study to derive from T cells and mast cells. We demonstrated additionally the expression in monocytes confirming the broad role of OSM in PN.^26^

Our data confirm a pathogenesis of PN most likely resulting from a complex neuroimmune interplay between neuronal receptors, various immune cells (notably Th2 cells) their cytokines, for example, IL‐4/IL‐13/IL‐31,^6^ as well as Th1 and Th17/22.^27^ In addition, we show Th17/Th22 expression to be significantly higher in lesional biopsies. While Th2 seems to be a key player in PN, the role of Th1/Th17/22 remains unclear and needs more clarification. Interestingly, IL‐17 can be associated to fibrotic skin diseases and thus also related to the pathogenesis in PN.^28^ Interestingly, we found two Th2 immune signatures, one (*IL4R*, *CCL17* and *CCL22*), correlating positively with barrier genes *LOR*/loricrin and *PPL*/periplakin and regulatory genes *FOXP3* and *IL37* and the second (*IL13*, *CCL18* and *IL10*) with markers of general inflammation (*IL1B* and *MMP12*), and Th17/22 markers (*IL17A*, *PI3*, *S100A7/8/9/12*). Markers within these correlation clusters generally correlated negatively with each other, highlighting the nuances of the Th2 pathway in this disease. The varied relationship between components of the Th2 axis and markers of other immune pathways, negative regulators and barrier markers may reflect both Th2's role in perpetuating further inflammation and barrier defects^29^ and a compensatory response to Th2 agonism.

The pathogenesis of PN may share immune similarities with AD, which is supported by the efficacy of (IL‐4/IL‐13‐targeting) dupilumab in both diseases.^19^, ^30^, ^31^ As in AD, our observed Th17/Th22 increase in PN may follow that of Th2 rather than act as key drivers of the disease.^29^ Correlation analysis of gene expression profiles and clinical NRS scores highlighted strong positive associations within two distinct groups of Th2 markers and other immune and barrier genes. NRS was significantly and positively correlated with Th2‐associated *IL10*, which is notable as *IL10* is related to the expression of monocytes and Th2 cells and helps regulate the immune response. Findings of this study affirm findings from previous transcriptomic studies,^2^, ^32^ as our results indicate that the Th1 and Th17/22 pathways, in addition to canonical Th2 pathway, play a role in PN.

Limitations of this study include small sample size and heterogeneity of the cohort. Nevertheless, the number of cases was defined previously based on prior studies^15^ and a contingency of additional patients was included to compensate for possible dropouts and a possible higher cohort variability. As no precise preliminary data were available for an exact calculation of a minimum number of cases, all analyses conducted here are descriptive and exploratory. Since there were no established definitions for PN at the time of this study (2017), diagnosis of PN was done at the discretion of the investigators. However, all investigators were experienced in diagnosing and managing PN; thus, all patients demonstrated characteristics typical of PN, and biomarkers were found to be generally similar across this heterogenous population with various etiologies. Another limitation is lack of exact discrimination of skin‐infiltrating immune cells in the immunohistochemical analysis, as the precise source of marker expression was not part of this study.

Overall, this cohort of patients with PN enrolled in the LOTUS‐PN study displayed a high itch burden with an associated sleep disruption at baseline. Biomarker analyses confirmed the presence of various biomarkers relevant to PN pathophysiology, such as IL‐31 and OSM and their receptors. These findings support the role of Th2 cytokines in PN.

## AUTHOR CONTRIBUTIONS

Conceptualization: SS, EGY, GY, DM and JFP. Data Curation: SS, EGY and JFP. Formal Analysis: SS, HW, DM, EGY, MK and EDD. Funding Acquisition: JFP. Investigation: HW and EDD. Project Administration: SS and EGY. Resources: SS, EGY and JFP. Supervision: SS, EGY and JFP. Visualization: HW, MK and EDD. Writing – original draft: SS, EGY, GY and JFP. Writing – review and editing: SS, EGY, GY, HW, DM, MK, EDD, KB and JFP.

## FUNDING INFORMATION

Kiniksa Pharmaceuticals provided financial support for the conduct of the clinical trial and preparation of the article. The sponsor played a role in the design of the clinical trial in addition to the collection, analysis and interpretation of the data, the writing of the manuscript and the decision to submit the article for publication.

## CONFLICT OF INTEREST STATEMENT

S. Ständer has received consulting and advisory board fees from AbbVie, Almirall, Beiersdorf, Bellus Health, Benevolent, Bionorica, Cara, Clexio, DS Biopharma, Eli Lilly, Galderma, Integrity CE, Kiniksa, Klinge Pharma, Leo Pharma, Pfizer, Sanofi, TouchIME, Trevi, Vifor and WebMD; speaker fees from Almirall, BMS, Eli Lilly, Sanofi, Galderma, L'Oréal, Omnicuris, Beiersdorf, Leo Pharma, Novartis, P.G. Unna Academy, Pfizer, UCB and Vifor; and research grants from Almirall, German Research Foundation (DFG), European Academy of Dermatology and Venereology (EADV), Leo Pharma, Novartis, Sanofi and Trevi Therapeutics. S. Ständer also reports conflicts of interest regarding dupilumab, aprepitant and difelikefalin. E. Guttman‐Yassky has received consulting fees from AbbVie, Almirall, Amgen, Arena, Asana Biosciences, Aslan Pharmaceuticals, AstraZeneca, Biolojic Design Ltd., Boehringer Ingelheim, Bristol Meyers Squibb, Cara Therapeutics, Celgene, Connect Pharma, DBV Technologies, Eli Lilly, EMD Serono, Evidera, Galderma, Gate Bio, Genentech, Ichnos Sciences, Incyte, Inmagene, Janssen Biotech, Kyowa Kirin, Leo Pharma, Merck, Pandion Therapeutics, Pfizer, Q32 Bio Inc., RAPT Therapeutics, Regeneron Pharmaceuticals, Inc., Sanofi, SATO Pharmaceutical, Siolta Therapeutics, Target Pharma Solutions, UCB and Ventyx Biosciences and grants or contracts from Amgen, AnaptysBio, Asana Biosciences, AstraZeneca, Boehringer Ingelheim, Cara Therapeutics, Celgene, Eli Lilly, Innovaderm, Kyowa Kirin, Leo Pharma, Novartis, Pfizer and Regeneron Pharmaceuticals. G. Yosipovitch is an advisory board member for Bellus Health, Eli Lilly, Galderma, GSK, Kiniksa Pharmaceuticals, LEO Pharma, Novartis, Pfizer, Regeneron Pharmaceuticals, Inc., Sanofi, Trevi Therapeutics, Celldex, Arcutis and AbbVie; has received grants/research funding from Kiniksa Pharmaceuticals, LEO Pharma, Novartis, Pfizer, Sanofi Regeneron, Escient and Eli Lilly; and is an investigator for Regeneron Pharmaceuticals, Inc., Sanofi, Galderma, Celldex and Kiniksa. E. Del Duca has received funding from the American Skin Association (ASA) Milstein Research Scholar Award Atopic Dermatitis. D. Metze reports travel support for Jahrestagung Arbeitsgemeinschaft Dermatologische Histologie (ADH) 2022 Annual Meeting and management of the International Dermpath Examination. M. Kim reports having received a research stipend from 2022 to 2023 from National Institutes of Health (NIH). K. Bondensgaard was an employee of Kiniksa Pharmaceuticals Corp. and owned stock in the company at the time of the study. J.F. Paolini is an employee of Kiniksa Pharmaceuticals Corp. and owns stock in the company. He is also an inventor on patent applications related to methods of using vixarelimab for treating skin diseases and disorders, including PN (patent application no. PCT/IB2019/000619). H.W. states no conflict of interest.

## ETHICAL APPROVAL

On 21‐Jun‐2017, Sterling Institutional Review Board (central IRB) approved the study protocol, informed consent form, patient questionnaires and patient‐facing documents. Where applicable, individual local IRBs and Ethics Committees (ECs) also reviewed and approved the protocol, informed consent form, patient questionnaires and patient‐facing documents for each site prior to the initiation of study activity at a site.

## ETHICS STATEMENT

The authors acknowledge adherence to the ICJME Recommendations for patient privacy. Specifically, that patients have a right to privacy that should not be violated without informed consent. Identifying information, including names, initials or hospital numbers, should not be published in written descriptions, photographs or pedigrees unless the information is essential for scientific purposes and the patient (or parent or guardian) gives written informed consent for publication. Informed consent for this purpose requires that an identifiable patient be shown the manuscript to be published. Authors should disclose to these patients whether any potential identifiable material might be available via the Internet as well as in print after publication.

## Supporting information


Table S1.

Figure S1.

Figure S2.

## Data Availability

Data will be made available upon reasonable written request to the corresponding author from researchers whose proposed use of the data for a specific purpose has been approved. The mechanism by which the data will be made available will be evaluated at the time of the request. Data will not be provided to requesters with potential or actual conflicts of interest, including individuals requesting access for commercial, competitive or legal purposes. Data access may be precluded for studies in which clinical data were collected subject to legal, contractual or consent provisions that prohibit transfer to third parties. All those receiving access to data will be required to enter into a Data Use Agreement (DUA), which shall contain terms and conditions that are customary for similar agreements and similar companies in the industry.
