# Supplementary material for: Th2 mRNA gene expression analysis separates Prurigo nodularis into two immune signature groups
Source: J Eur Acad Dermatol Venereol. 2025 Jul 2;39(10):1750–9. doi: 10.1111/jdv.20812 (PMC12466102; doi:10.1111/jdv.20812)
Supplement: Supplementary file 1 — Table S1. [file JDV-39-1750-s003.docx]

**Table S1.** Primers and Probes for Taqman Low Density Array

| **18S** | Hs99999901_s1 |
| --- | --- |
| **CCL11** | Hs00237013_m1 |
| **CCL13** | Hs00234646_m1 |
| **CCL17** | Hs00171074_m1 |
| **CCL18** | Hs00268113_m1 |
| **CCL20** | Hs00355476_m1 |
| **CCL22** | Hs01574247_m1 |
| **CCL26** | Hs00171146_m1 |
| **CCL5** | Hs00982282_m1 |
| **CXCL1** | Hs00236937_m1 |
| **CXCL10** | Hs00171042_m1 |
| **CXCL2** | Hs00601975_m1 |
| **CXCL9** | Hs00171065_m1 |
| **FLG** | Hs00856927_g1 |
| **FOXP3** | Hs01085834_m1 |
| **IFNG** | Hs00989291_m1 |
| **IL10** | Hs00961622_m1 |
| **IL13** | Hs00174379_m1 |
| **IL15** | Hs01003716_m1 |
| **IL17A** | Hs00174383_m1 |
| **IL1B** | Hs01555410_m1 |
| **IL2** | Hs00174114_m1 |
| **IL22** | Hs01574154_m1 |
| **IL23A** | Hs00372324_m1 |
| **IL31** | Hs01098710_m1 |
| **IL31RA** | Hs00371172_m1 |
| **IL32** | Hs00992441_m1 |
| **IL37** | Hs00367201_m1 |
| **IL4** | Hs00174122_m1 |
| **IL4R** | Hs00965056_m1 |
| **IL5** | Hs00174200_m1 |
| **IL6** | Hs00174131_m1 |
| **IL9** | Hs00914237_m1 |
| **JAK2** | Hs01078136_m1 |
| **KRT16** | Hs00955082_g1 |
| **LOR** | Hs01894962_s1 |
| **MMP12** | Hs00159178_m1 |
| **OSM** | Hs00171165_m1 |
| **OSMR** | Hs00384276_m1 |
| **PI3** | Hs00160066_m1 |
| **PPL** | Hs00160312_m1 |
| **RPLP0** | Hs99999902_m1 |
| **S100A12** | Hs00942835_g1 |
| **S100A7** | Hs01923188_u1 |
| **S100A8** | Hs00374264_g1 |
| **S100A9** | Hs00610058_m1 |
| **STAT1** | Hs01013996_m1 |
| **STAT3** | Hs00374280_m1 |
