# Supplementary material for: Th2 mRNA gene expression analysis separates Prurigo nodularis into two immune signature groups
Source: J Eur Acad Dermatol Venereol. 2025 Jul 2;39(10):1750–9. doi: 10.1111/jdv.20812 (PMC12466102; doi:10.1111/jdv.20812)
Supplement: Supplementary file 2 — Figure S1. [file JDV-39-1750-s002.pptx]

## Slide 1
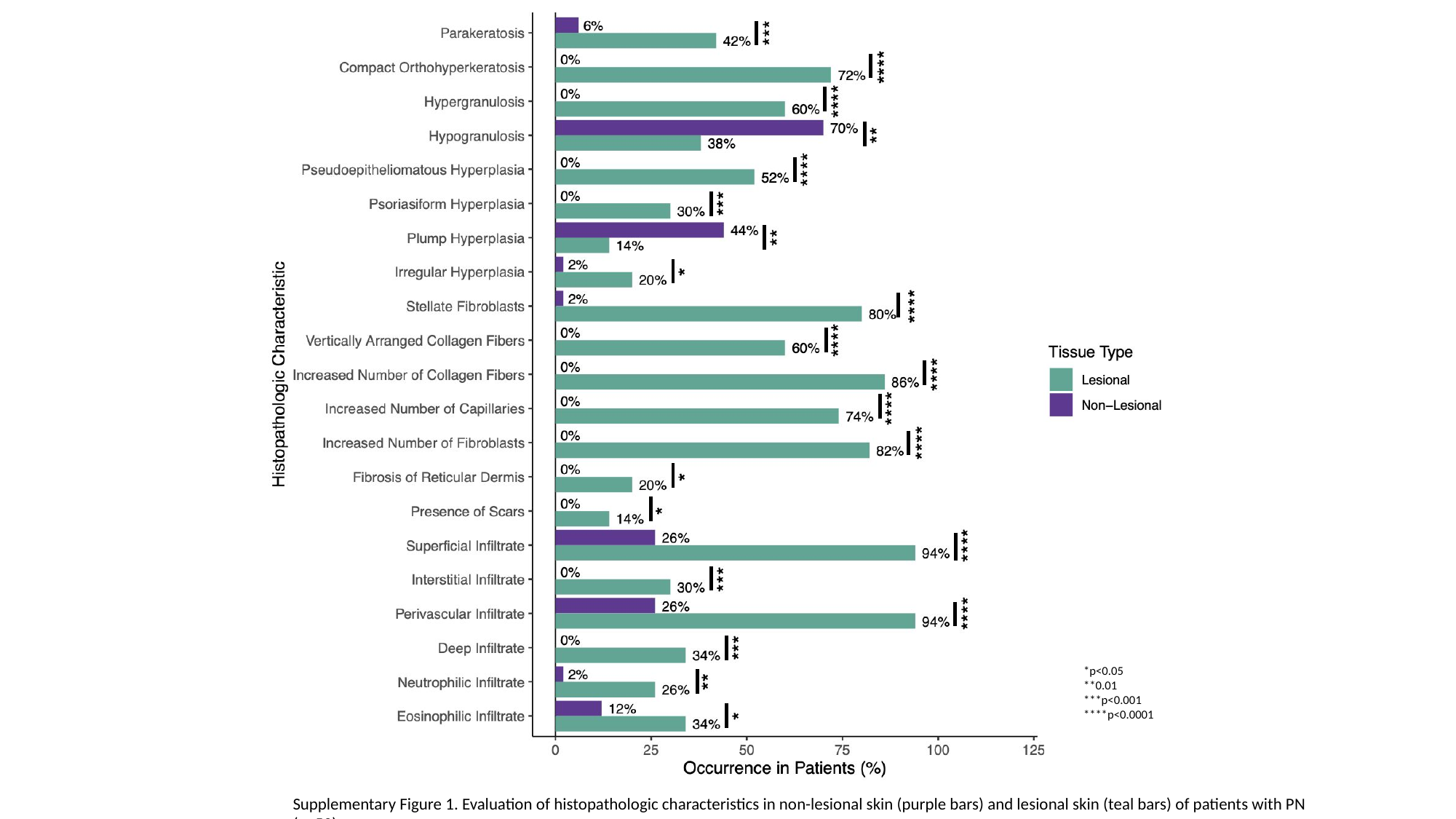

*p<0.05
**0.01
***p<0.001
****p<0.0001
Supplementary Figure 1. Evaluation of histopathologic characteristics in non-lesional skin (purple bars) and lesional skin (teal bars) of patients with PN (n=53).
