# Supplementary material for: Th2 mRNA gene expression analysis separates Prurigo nodularis into two immune signature groups
Source: J Eur Acad Dermatol Venereol. 2025 Jul 2;39(10):1750–9. doi: 10.1111/jdv.20812 (PMC12466102; doi:10.1111/jdv.20812)
Supplement: Supplementary file 3 — Figure S2. [file JDV-39-1750-s001.pptx]

## Slide 1
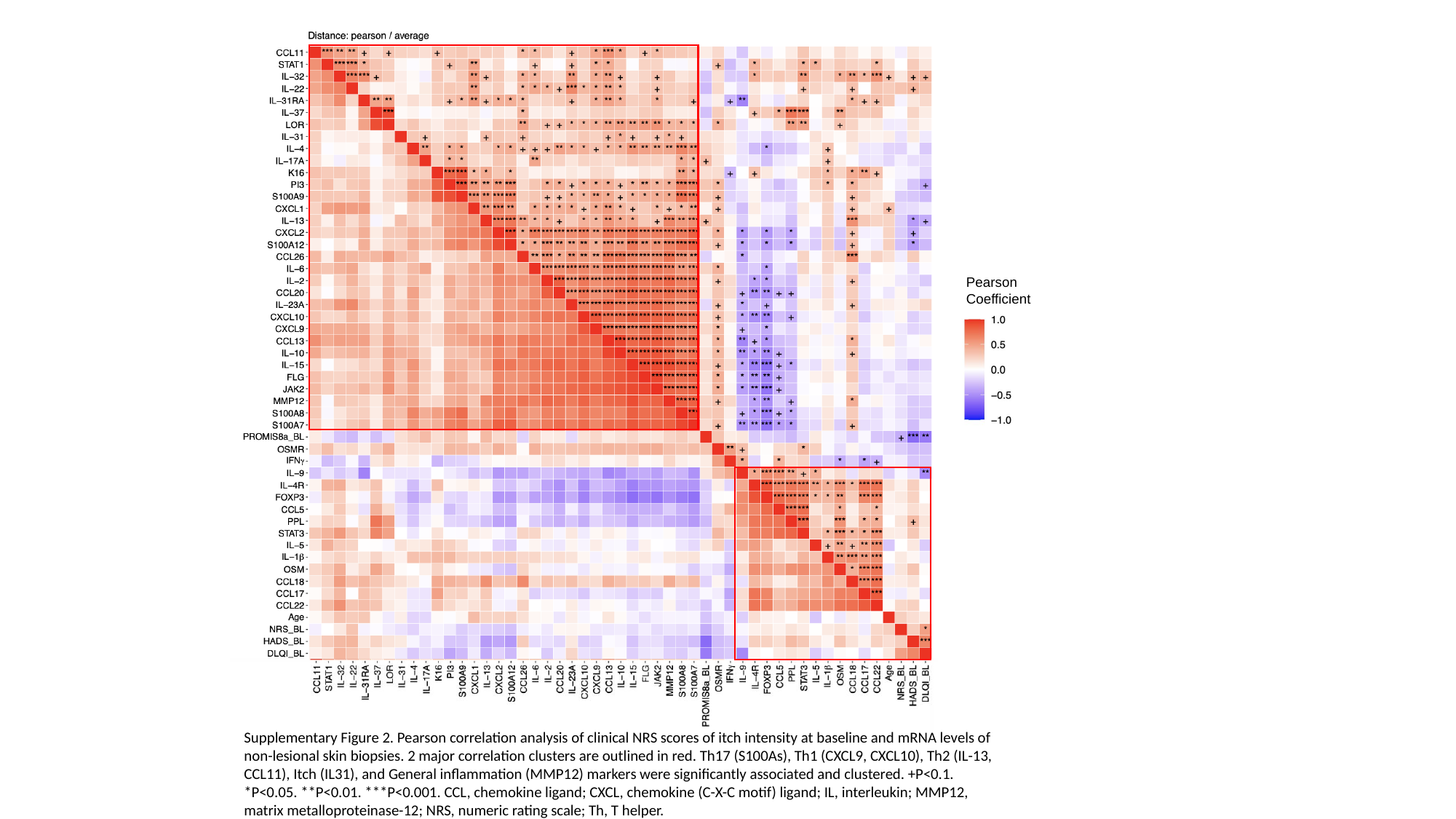

Pearson Coefficient
Supplementary Figure 2. Pearson correlation analysis of clinical NRS scores of itch intensity at baseline and mRNA levels of non-lesional skin biopsies. 2 major correlation clusters are outlined in red. Th17 (S100As), Th1 (CXCL9, CXCL10), Th2 (IL-13, CCL11), Itch (IL31), and General inflammation (MMP12) markers were significantly associated and clustered. +P<0.1. *P<0.05. **P<0.01. ***P<0.001. CCL, chemokine ligand; CXCL, chemokine (C-X-C motif) ligand; IL, interleukin; MMP12, matrix metalloproteinase-12; NRS, numeric rating scale; Th, T helper.
